# Outcomes of a Randomized Trial of a Cognitive Behavioral Enhancement to Address Maternal Distress in Home Visited Mothers

**DOI:** 10.1007/s10995-016-2125-7

**Published:** 2016-08-17

**Authors:** Elizabeth McFarlane, Lori Burrell, Anne Duggan, Darius Tandon

**Affiliations:** 1 Department of Population, Family and Reproductive Health, Johns Hopkins University Bloomberg School of Public Health, Baltimore, MD USA; 2 Northwestern University Fienberg School of Medicine, Chicago, IL USA

**Keywords:** Maternal stress, Depression, Prevention, Home visiting, CBT, Parent child interaction

## Abstract

*Objectives* To assess the effectiveness of a 6-week, cognitive behavioral therapy (CBT) group-based enhancement to home visiting to address stress and prevent depression as compared with home visiting as usual in low income mothers of young children. *Methods* We conducted a randomized controlled trial with 95 low-income mothers of young children to assess the effectiveness of a 6-week, cognitive behavioral group-based enhancement to Healthy Families America and Parents as Teachers home visiting (HV/CBT = 49) to address stress and prevent depression as compared with home visiting as usual (HV = 46). Booster sessions for the HV/CBT group were offered at 3 and 6 months. Participants completed measures of coping, stress and depression at three points: baseline prior to randomization, post-intervention, and 6 months post-intervention. Parent child interaction was also measured at 6 months. *Results* Intent-to-treat analyses found improved coping and reduced stress and depression post-intervention. While impacts on these outcomes were attenuated at 6 months, positive impacts were observed for selected aspects of mothers’ interactions with their children. Maternal characteristics at baseline were associated with participation in the intervention and with post-intervention and 6-month outcomes. Mothers with lower levels of stress and those with fewer children were more likely to attend intervention sessions. Mothers with lower levels of stress had more favorable post intervention outcomes. *Conclusions* CBT group-based enhancement to home visiting improved maternal coping, reduced stress and depression immediately post intervention but not at 6 months, suggesting more work is needed to sustain positive gains in low-income mothers of young children.

## Significance

Low-income mothers are at elevated risk for poor mental health. The negative effects of poor maternal mental health on child development are well documented. This study extends the knowledge on CBT effectiveness in addressing depression in low-income home visited mothers and provides support for its impact on parenting behavior.

## Introduction

Prenatal and early life experiences affect early development and set the trajectory for health and well-being across the life span (National Research Council and Institute of Medicine 2000). Many children are born into isolated, vulnerable families whose multiple stressors give rise to an inhospitable environment for the critical first years of development (Felitti et al. 1998; National Research Council and Institute of Medicine 2000, 2009). Maternal distress defined as depression, anxiety and/or perceived stress adversely influences parent–child interaction and parenting in general. Prevalence of depressive symptoms of 17.1–23.1 % have been reported in new mothers (Cox et al. 1987). Prevalence of depressive symptoms in at-risk women is nearly twice that of their low risk counterparts (Mayberry et al. 2007); underscoring the need for prevention, particularly in populations with elevated risk for distress (Ammerman et al. 2010; Pawlby et al. 2008).

Home visiting is a strategy to engage and improve outcomes of at-risk families (Avellar et al. 2013; Martin et al. 2008). Healthy Families America (HFA) and similar home visiting models enroll participants with psychosocial risks, including maternal stress and depression with the intent of reducing risks for these mothers (Avellar et al. 2013; Michalopoulos et al. 2013). Moderation of program impacts as a result of maternal psychosocial characteristics is common (McFarlane et al. 2014). Yet, research shows that home visiting program impacts are modest (Avellar et al. 2013; Sweet and Applebaum 2004). Implementation science provides a theoretical framework and methods for understanding the reasons for this (Durlak and Dupre 2008; Fixsen et al. 2005). In short, one must look at the theory of change and determine if the actual services as delivered align with the organizational- and individual-level factors for how services should be delivered (Segal et al. 2012). Research on the services provided by HFA home visitors confirms that home visitors often failed to address the stressors and stress-related behaviors common in enrolled families. For example, rates for home visitor discussion of poor mental health in mothers who scored positive for depression and anxiety were only 15 and 45 % in two statewide studies (Duggan et al. 2004, 2007).

Our earlier research found that home visiting models and implementation systems were ‘under-developed’ with regard to addressing maternal stress and distress (Duggan et al. 2004, 2007). Thus, while home visitors were provided explicit performance goals and curricula for promoting positive parenting, they had far less guidance and support in ways to address maternal stress. Home visitors gave lower ratings of the training they received for addressing parenting risks than for their training to promote child development (Duggan et al. 2011). They felt less competent to address maternal stress than to promote positive parenting. As a result, they were less likely to attend to the stress-reduction aspects of their role than to activities that focused more directly on parenting behavior (Duggan et al. 2004, 2007, 2011; Tandon et al. 2005).

This suggests that home visiting programs must more clearly define their service models to reduce maternal stress. This means spelling out clearly and concisely the intended program services and mediating outcomes for reducing maternal distress and the specific role of each staff member in this regard. Beyond this, programs must develop implementation systems that predispose, enable and reinforce staff members to carry out their defined roles. This means providing adequate training, supervision, protocols, community links, and administrative supports for staff to identify and work with families in this regard (Michalopoulos et al. 2013).

One key step is to be specific in what we mean by ‘stress.’ This term is used in many different ways, but most definitions incorporate a process in which environmental demands exceed an individual’s adaptive capacity, resulting in psychological or biological changes that adversely affect health (Cohen et al. 1995). This process can be thought of as having three main components: (1) environmental stressors; (2) the individual’s appraisals or perceptions of stress; and (3) the individual’s stress responses, including affective, behavioral and biologic responses. Thus, home visiting programs could reduce maternal distress in several different ways, ranging from reducing environmental stressors to promoting more favorable evaluations of stress to promoting more effective behavioral responses to stress.

In this study we sought to determine the effectiveness of a group-based enhancement to usual home visiting services to address mothers’ appraisals of stress and affective and behavioral responses to stress. We sought to reduce maternal stress and depression, and improve maternal coping and parent child interaction. Our approach was to enhance the home visiting service model and implementation system by integrating a 6-week cognitive-behavioral intervention into usual home visiting services. We hypothesized that mothers randomly assigned to the enhancement would have better coping, reduced stress and depression, and more sensitive and responsive interactions with their children. We also explored the role of maternal baseline characteristics on intervention attendance and outcomes. We hypothesized that intervention participation and outcomes would vary based on maternal baseline characteristics, with mothers experiencing less stress garnering greater program benefits.

## Methods

### Description of the Intervention to Reduce Maternal Stress and Depression

Together, through a research- to-practice partnership between the Hawaii Department of Health, three community-based home visiting providers and Johns Hopkins University, we conducted a review of the literature to identify evidence-based interventions to address maternal stress and depression (McFarlane et al. 2014). The team chose the Mothers and Babies Course (MB) (Muñoz et al. 2007) as the home visiting enhancement because of evidence of effectiveness, its group-based format, and perceived fit with the organizational capacity of the participating service providers. The team committed to testing its impact rigorously. MB is a cognitive-behavioral group intervention developed for low-income and minority perinatal populations faced with multiple stressors. The MB integrates proven cognitive-behavioral methods for managing stress and reducing psychosocial symptoms (Lewinsohn et al. 1992).

The MB Course was implemented following the protocol developed by Tandon and colleagues (Tandon et al. 2011). MB was delivered concurrently with home visiting services by masters-level trained clinical specialist at each site. The clinical specialist and home visitors were trained in-person by a model developer and the study director. The research team and service providers reviewed the MB Course and modified the curriculum to increase cultural relevance for mothers by using names, phrases, and terminology common to the local population. Mothers in MB received 6 weekly 2-h intervention sessions in a group format lead by each home visiting agency’s clinical specialist. Group sessions were held at the offices of the service provider. Weekly reinforcement of core topics by home visitors trained on the MB Course were provided to mothers in the MB group during home visits. Each session had both didactic instruction on core content, activities, and group discussion. The course was divided into three two-session modules: pleasant activities, thoughts, and contact with others. These modules mapped onto core cognitive-behavioral approaches for the treatment and prevention of depression and stress reduction. Between group sessions, at home visits, home visitors provided 5–10 min of one-on-one reinforcement of key content covered during group sessions.

### Study Sites and Participants

The project was carried out with three home visiting program sites in Hawaii from 2012 to 2014. The home visiting programs enrolled eligible mothers at the birth of their child through standardized assessment protocols to identify eligible mothers on the basis of demographic and psychosocial attributes associated with poor parenting, such as poverty, inexperience, limited education, intimate partner violence and poor mental health. Enrolled mothers receive services by a paraprofessional home visitor to reduce family risk through the provision of emotional support, encouragement to seek professional counseling and treatment when needed, teaching parents about infant/child care and development, the importance of positive interaction, and by fostering mothers’ problem solving skills.

Home visited women were eligible for the study if they were 18 years or older, spoke English, were not on creative outreach (defined as not yet fully enrolled in program services), and were free from work or school on the days/time the MB Course was offered. Additionally, because MB is intended to prevent rather than treat major depression, those mothers known to have major depression were excluded from the study. The study sample (n = 95) was enrolled in 7 cohorts over a 12 month period. Randomization of mothers was stratified by agency and occurred in cohorts to address the constraints of the MB group limit of 10 participants and the availability of clinical specialist to conduct the groups. Mother–child dyads were identified based on the child born during the study period and for whom program eligibility was determined.

**Fig. 1:**
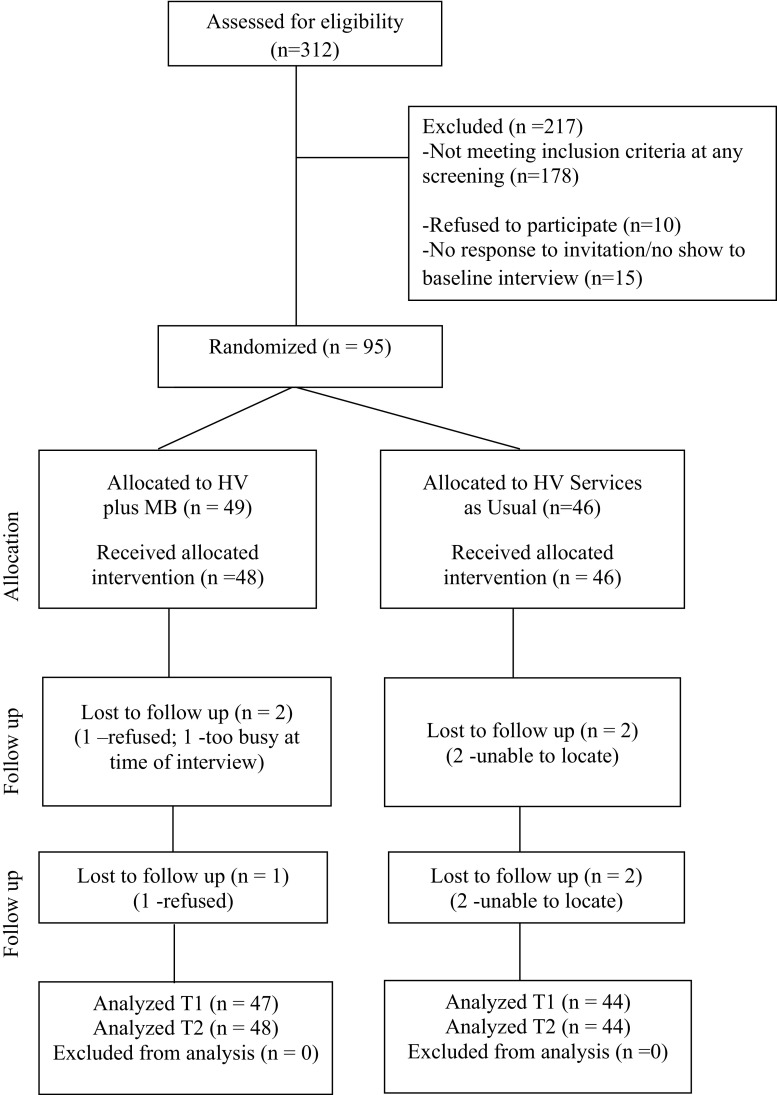
Study CONSORT diagram

As seen in Fig. 1, of the 312 mothers identified as enrolled in home visiting during the 12 months of study recruitment, 217 were excluded from the study. Overall, 178 mothers (57 % of identified home visiting enrollees) were excluded because they did not meet study eligibility criteria (e.g. fully enrolled in home visiting, work and school scheduled allowed for group participation) and 29 (22 %) were excluded because they could not be reached. Of the 105 mothers who were reached and eligible, 95 (90 %) agreed to participate and completed the baseline interview while the other 10 (10 %) refused. Follow-up rates for mothers enrolled in the study exceeded 95 %. Retention was similar across study groups.

### Description of Measures

Research staff collected written study consent and data via in-person maternal interview at baseline and two follow-up points, immediately post intervention and at 6 months post intervention. Interviews were conducted in the mother’s home or a location of her choosing. Research interviewers were not involved in the randomization of participants or informed of the mother’s group assignment.

### Process Measures

The clinical specialists providing the MB Course documented each mother’s attendance. A high level of attendance was defined as attending 5 or more of the group sessions. Participation in MB was defined as attending at least 1 group session.

### Maternal Psychosocial Measures

The Beck Depression Inventory-II (BDI-II) is a 21-item self-report inventory with multiple choice response options scored from 0 to 3 (Beck et al. 1996). The BDI–II assesses the intensity of depression in clinical and normal patients. Each item has a list of four statements arranged in increasing severity about a particular symptom of depression. Higher scores indicate more severe depressive symptoms. Cut score guidelines may be adjusted based on the characteristics of the sample, and the purpose for use of the BDI-II. We used a cut point of 19 for moderate and 30 for severe depressive symptoms. Cronbach’s alpha of .92 was reported for out-patient populations (Beck et al. 1996).

The Ways of Coping (WOC) Questionnaire identifies the thoughts and actions an individual has used to cope with a specific stressful encounter in the last week. It measures coping processes, not coping dispositions or styles. The 8 Scales/Coping Factors include: Confrontive Coping; Distancing; Self-Controlling; Seeking Social Support; Accepting Responsibility; Escape-Avoidance; Planful Problem Solving; and Positive Reappraisal. Individuals respond to each item on a four-point Likert scale, indicating the frequency with which each strategy is used (Folkman and Lazarus 1985). Factor-based reliability estimates, from several studies reporting WOC data, averaged .73 and ranged from .56 to .85 (Edwards and O’Neill 1998).

The Life Experience Survey (LES) is a self-report measure of positive and negative events experienced over the previous year and the perceived stress associated with those events. The instrument includes 60 items divided into two sections. Section 1 contains 50 life changes that are common to individuals in a wide variety of situations. Section 2 contains 10 items that are for students only. Respondents rate each life event experienced on a 7-point scale ranging from −3 (extremely negative) to +3 (extremely positive). Reliability coefficient of .64 has been reported for the LES total score (Sarason et al. 1978).

The Perceived Stress Scale (PSS) is a 10 item instrument designed to measure the degree to which situations in one’s life are appraised as unpredictable, uncontrollable and stressful (Cohen et al. 1983, Cohen and Williamson 1988). Response options are on a 5-point scale ranging from 0 (never) to 4 (very often). Responses to the 10 items are summed to create a psychological stress score, with higher scores indicating greater psychological stress. Cronbach’s alpha of .91 was reported in two US samples. The Perceived Stress Scale is not a diagnostic instrument; there are no score cut-offs. Cohen recommends comparisons within your own sample. For this study, continuous and binary variables were used in analyses. For the binary variable we dichotomized the sample into mothers with low and high levels of baseline stress at the actual mean, with scores ≥16 indicating high baseline stress.

### Parent Child Interaction

The Keys to Interactive Parenting Scale (KIPS) is a video-taped observational measure that assesses the quality of 12 parenting behaviors that research has shown to be linked to children’s healthy development (Comfort and Gordon 2006). The 12 items are set on 5-point scales with behavioral descriptions at the odd points of 1, 3 and 5. A rating of 5 is the most favorable. Individual item scores and total means score may be calculated. Comfort and Gordon (2006) report internal consistency (alpha .89) and inter-rater reliability (.90–.96) for the KIPS. Raters for this study completed on-line training for certification and were blind to treatment condition.

The 12 KIPS Parenting Behaviors include: Sensitivity of Responses; Supports Emotions; Physical Interaction; Involvement in Child’s Activities; Open to Child’s Agenda; Language Experience; Reasonable Expectations; Adapts Strategies to Child; Limits and Consequences; Supportive Directions; Encouragement; and Promotes Exploration/Curiosity. To our knowledge no prior studies of the MB Course included the KIPS. Thus, we considered its inclusion as an exploratory extension of the impact of MB. We did not expect the Mothers and Babies Course to influence all parenting behaviors. Our review of the MB content and theory of change suggested the course would improve ratings of the following: S*ensitivity of Responses, Physical Interaction, Involvement in Child’s Activities, Reasonable Expectations* and *Encouragement*.

This study was reviewed and approved by the Johns Hopkins University Institutional Review Board and, when present, the review boards of the participating community providers.

### Analysis

All analyses were run with SPSS Statistical Software Version 23. Data was complete for all measures and all participants except where the participant declined to participate in the observational measure of parent–child interaction (n = 3) or declined the follow-up interview completely (n = 3). Intent-to-Treat (ITT) effects were calculated for MB versus home visiting services as usual. We investigated whether maternal baseline characteristics were associated with participation in the MB Course. Student’s *t* test and Chi square were used to assess the baseline comparability of the treatment groups overall and by MB participation level. Linear and logistic regressions were used to assess the impact of the intervention on stress, coping, depression and maternal child interaction. Tests of program impact controlled for baseline measures of outcomes as well as baseline covariates on which the intervention and control groups included in the analysis differed significantly (*p* < .05). We tested for program effects among subgroups defined by baseline covariates found to be significantly associated with level of participation in mothers assigned to MB.

## Results

### Maternal Baseline Characteristics

Maternal baseline characteristics are reported in Table 1. The mean age of mothers in the study was 27.6 (SD = 6.4) years. Twenty-four percent had less than a high school education and for thirty percent the intervention child was their first born. Sixty-six percent of the mothers were married or living with the father of their child. Most mothers were Asian and Pacific Islander (72 %) and most lived in households below poverty level. The MB and control mothers were similar on all baseline characteristics except the proportion of mothers for whom the focal child was the first born, 18 and 43 %, respectively (*p* = .01).

**Table 1:** Maternal baseline characteristics, overall and by study group (n = 94)

	Overall (n = 94)	MB (n = 49)	Control (n = 45)	*p*
Mom age [mean (SD)]	27.6 (6.4)	28.0 (6.6)	27.1 (6.4)	.52
Child age in months [mean (SD)]	14.0 (11.9)	14.6 (12.6)	13.3 (9.9)	.58
Highest education completed				
Less than HS graduate	24 %	26 %	21 %	.70
High school graduate	32 %	30 %	35 %	
Beyond HS	44 %	44 %	44 %	
Target child is first born	**30** **%**	**18** **%**	**43** **%**	**.01**
Married/living with father of baby	66 %	65 %	67 %	.89
Maternal primary race				
AAPI	72 %	73 %	72 %	
White	8 %	6 %	9 %	.98
Multiracial	20 %	20 %	20 %	
Below poverty level	72 %	73 %	72 %	.95
Perceived Stress Scale	17.0 (7.0)	17.6 (7.6)	16.2 (6.3)	.34
BDI depression	12.7 (10.1)	13.1 (10.7)	12.3 (9.4)	.72
BDI Mod/severe depression	25 %	26 %	23 %	.67

Significant results (*p* < .05) and trends (*p* < .10) are given in bold

Cases with completed baseline and at least 1 follow-up (post-intervention and/or 6 mo interview)

Mothers in the MB and control groups were similar in their psychosocial risks at baseline. Overall mean scores for stress (PSS) and depression (BDI) were 17.0 (SD = 7.0) and 12.7 (SD = 10.1), respectively. About a quarter of mothers in each group scored in the moderate to severe range on the BDI.

### Attendance in Mothers and Babies

A quarter of mothers assigned to the treatment condition did not attend a single session. Another 10 % of the sample attended 1–4 of the group sessions. Most mothers (63 %) attended five or more of the sessions. Differences in attendance by agency were noted but didn’t reach statistical significance. The percent of mothers with a high level of attendance (attended 5 or more group sessions) ranged from 53 to 86 % across the three agencies. Mothers with a high level of attendance differed from mothers with lower attendance on two characteristics at baseline. High dose mothers reported lower levels of stress (*p* < .05) and had fewer children (*p* < .05).

### Overall Post-intervention and 6 Month Maternal Psychosocial Outcomes

Post Intervention and 6 Month Follow-up ITT findings are shown in Table 2. Significant positive post intervention impacts for the MB group were found for maternal coping, depression and stress. Increased coping on the Accepting Responsibilities subscale (*p* < .01) of the Ways of Coping Scale was found for the MB group. Decreased scores on the Beck Depression Inventory (*p* < .05) and Perceived Stress Scale (*p* < .06) were also reported for the MB group. Effect sizes for these findings were (d = .55, d = .38 and d = .35) respectively, suggesting moderately strong treatment effects for these outcomes. No differences were seen at post-intervention on the Life Events Scale. The maternal psychosocial impacts were attenuated at 6 months with no significant differences found for any maternal psychosocial outcome.

**Table 2:** Program impact on maternal psychosocial outcomes at post intervention and 6 month follow-up

	Post intervention (n = 91)					6 months (n = 92)				
	MB (n = 47)	Control (n = 44)	B	95 % CI	*p*	MB (n = 48)	Control (n = 44)	B	95 % CI	*p*
Ways of coping										
Confrontive coping	6.84	6.89	−0.05	−1.8, 1.7	.95	6.63	6.30	0.33	−1.3, 2.0	.70
Distancing	7.23	6.51	0.72	−0.9, 2.3	.38	6.56	6.54	0.01	−1.5, 1.5	.99
Self-controlling	9.30	8.28	1.02	−0.7, 2.8	.25	8.08	9.18	−1.10	−2.8, 0.6	.19
Seeking social support	8.51	7.99	0.51	−1.2, 2.2	.55	7.75	8.25	−0.50	−2.3, 1.3	.59
Accepting responsibility	**5.09**	**3.50**	**1.59**	**0.5**, **2.7**	<**.01**	4.02	3.88	0.14	−1.0, 1.26	.80
Escape/avoidance	7.22	6.71	0.50	−1.4, 2.4	.60	6.39	6.38	0.01	−1.8, 1.8	.99
Planful problem solving	10.20	8.95	1.24	−0.4, 2.9	.14	9.55	10.02	−0.47	−2.2, 1.2	.58
Positive reappraisal	10.85	10.19	0.66	−1.3, 2.6	.51	10.32	9.48	0.83	−1.2, 2.9	.42
Beck’s Dep. inventory										
Total score	**8.62**	**11.87**	−**3.25**	−**6.6**, **0.1**	**.05**	10.81	10.57	0.24	−3.8, 4.3	.91
% w/Mod. or Sev. Dep^a^	17 %	20 %	0.78	0.2, 2.6	.69	19	19	0.96	0.3, 3.1	.94
Perceived Stress Scale										
Total score	**14.54**	**16.82**	−**2.28**	−**4.7**, **0.1**	**.06**	14.70	15.96	−1.26	−4.0, 1.5	.37
Life events										
Total affect score	2.41	1.56	0.85	−1.9, 3. 6	.53	2.82	0.27	2.55	−1.1, 6.2	.17

Significant results (*p* < .05) and trends (*p* < .10) are given in bold

Models control for program site, TC is first born, and baseline measure of dependent variable. Means reported in table are adjusted

^a^Adjusted odds ratio

## 6 Month Parent Child Interaction Outcomes

Positive impacts were found for two of the five aspects of parent child interaction for which we anticipated program effects (Table 3). A significant positive result was obtained on the Sensitivity of Responses (*p* < .04) and a positive trend on the Involvement in Child’s Activities (*p* < .07) items of the KIPS. Effect sizes for these findings were (d = .50 and d = .52) respectively, suggesting moderately strong treatment effects for these outcomes.

**Table 3:** Program impact on parent child interaction at 6 month follow-up

	6 months (n = 89)				
	MB (n = 46)	Control (n = 43)	B	CI	*p*
Keys to Interactive Parenting Scale					
Sensitivity of responses	**3.82**	**3.34**	**0.49**	**0.0**, **0.9**	**.04**
Physical interaction	4.09	3.89	0.20	−0.2, 0.6	.39
Involvement in child’s activities	**4.02**	**3.62**	**0.40**	−**0.03**, **0.8**	**.07**
Reasonable expectations	3.58	3.53	0.05	−0.4, 0.5	.85
Encouragement	4.00	3.92	0.08	−0.4, 0.5	.72

Significant results (*p* < .05) and trends (*p* < .10) are given in bold

Models control for program site and first born child. Means reported in table are adjusted

### MB Effects at Post Intervention and 6-Month Follow-Up by Maternal Baseline Stress

To explore how maternal baseline stress may have influenced program impact on outcomes, we dichotomized the sample into mothers with low and high levels of baseline stress. The regression analyses showed that the positive impacts observed for the full sample at post-intervention were evident for MB vs. control mothers with lower levels of stress (Table 4). Additional positive impacts emerged for the MB mothers with low baseline stress on the Ways of Coping subscales: Self-Controlling (B 2.69, *p* < .05) and Planful Problem Solving (B 2.88, *p* < .05). These impacts were similar to those reported in the ITT analyses at post-intervention (Table 4).

**Table 4:** Program impact on maternal psychosocial outcomes at post intervention and 6 months by baseline maternal stress

	Post intervention (n = 91)						6 months (n = 92)					
	Low stress (n = 45)			High stress (n = 46)			Low stress (n = 45)			High stress (n = 47)		
	B	95 % CI	*p*	B	95 % CI	*p*	B	95 % CI	*p*	B	95 % CI	*p*
Ways of coping												
Confrontive coping	.68	−1.5, 2.8	.53	−1.00	−3.8, 1.8	.73	−1.01	−3.2, 1.2	.63	1.15	−1.4, 3.7	.37
Distancing	1.34	−.98, 3.7	.25	−.26	−2.8, 2.3	.84	−.54	−2.4, 1.3	.56	.79	−1.5, 3.1	.49
Self-controlling	**2.69**	**.19**, **5.2**	**.04**	−.75	−3.5, 1.9	.58	−**2.32**	−**4.7**, **.05**	**.06**	.26	−2.3, 2.8	.84
Seeking social support	.41	−1.9, 2.7	.72	.02	−2.8, 2.8	.99	−1.58	−3.9, .75	.18	−.15	−3.2, 2.9	.92
Accepting responsibility	**2.47**	**1.1**, **3.8**	**.001**	.61	−1.1, 2.4	.48	−1.02	−2.4, .39	.15	1.02	−.72, 2.8	.24
Escape/avoidance	.44	−2.5, 3.4	.76	.64	−2.1, 3.4	.64	−1.37	−3.5, .77	.20	1.22	−1.7, 4.2	.41
Planful problem solving	**2.88**	**.59**, **5.2**	**.02**	−.84	−3.2, 1.5	.47	−1.85	−4.4, .65	.14	.74	−1.6, 3.0	.52
Positive reappraisal	1.25	−1.9, 4.4	.43	−.54	−3.3, 2.2	.69	−1.55	−4.3, 1.2	.27	**3.02**	**.12**, **5.9**	**.04**
Beck’s depression inventory												
Total score	−**3.77**	−**8.0**, **.44**	**.08**	−3.10	−8.6, 2.4	.27	−.70	−6.2, 4.8	.80	−.03	−6.1, 6.0	.99
Life events												
Total affect score	1.91	−1.8, 5.6	.31	.14	−4.0, 4.3	.95	.40	−4.2, 5.0	.86	**4.92**	−**.90**, **10.7**	**.10**

Significant results (*p* < .05) and trends (*p* < .10) are given in bold

Models control for program site, TC is first born, and baseline measure of dependent variable. Means reported in table are adjusted

There was a trend toward improved scores on the Beck Depression Inventory (B −3.77, *p* < .08) for MB mothers with lower levels of stress at baseline.

Maternal psychosocial outcomes at 6-month follow-up by baseline stress level were similar to those seen in the overall analyses with three exceptions. MB mothers with lower levels of stress had a greater reduction in scores on the Self Controlling subscale of the Ways of Coping (B −2.32, *p* < .10). MB mothers with higher levels of stress had more favorable scores on the Positive Reappraisal subscale of the Ways of Coping (B 3.02, *p* < .05) and a trend was seen on the Total Affect score of the Life Events Scale (B 4.92, *p* < .10).

## Discussion

Home visiting has received unprecedented attention in recent years. Funding through the Maternal, Infant, and Early Childhood Home Visiting (MIECHV) Program has increased nearly 50 % since 2011 (Children’s Budget 2015). The intent of the investment has been to support more parents and young children but also to broaden and strengthen impacts by improving service models and implementation systems. Maternal stress and depression’s high prevalence, adverse influence on parenting, and moderation of home visiting effects underscore the need to prevent and address these conditions within the context of home visiting (Ammerman et al. 2007; McFarlane et al. 2014; Tandon et al. 2011).

This study extends previous studies of Mothers and Babies in at least two ways. First, it examined engagement and impacts on outcomes for Asian and Pacific Islander mothers. Second, it included the quality of mother–child interaction as an outcome. In assessing parent child interaction we explored program impacts on the global ratings of individual items of the KIPS rather than using the over-all scale score as designed (Comfort et al. 2011). Our intent in doing so was to increase the sensitivity of measurement by focusing on the subset of items we felt would be influenced by the MB course.

Limitations of the study included the small sample size, and the use of self-report measures instead of structured diagnostic interviews or biomarkers to assess psychological distress and physiological stress. Additionally, we did not include a baseline measure of parent child interaction. Doing so may have strengthened study findings.

Strengths of the study include its randomized controlled design, assessment of outcomes at two points, 97 % follow-up of study participants and observational assessment of parent–child interaction. Additionally, this study was carried out under real-world conditions by home visiting program staff operating in three diverse communities in Hawaii.

Overall, this study suggests that mothers will participate in preventive group-based interventions to address maternal stress and depression offered as part of home visiting. Our findings also add to a growing body of evidence that group cognitive-behavioral models can reduce the burden of stress and depression experienced by home visited mothers. Additionally, the positive gains in parent–child interaction extends and strengthens prior research on the MB and suggests that potential cost benefits may emerge through improved child health and development across the life course. The parent child interaction findings of improved maternal sensitivity are particularly salient given the enduring effects maternal sensitivity has on social and academic competence from childhood to adolescence and adulthood (Cassidy 1994; Fraley et al. 2013; Sroufe et al. 2005; Raby et al. 2015).

## Conclusions

The study confirmed what previous research has reported: maternal stress and depression are common in home visited women. Our work showed that an enhancement to home visiting services to address maternal stress and prevent depression resulted in short-term post intervention improvements in coping and reduced stress and depression.

The Mothers and Babies Course increased coping, reduced perceived stress and depression post-intervention. These gains in coping and reductions in stress and depression were attenuated at 6 months. However, mothers in the MB Course had more sensitive responses to and increased involvement with their children on an observational measure of parent child interaction at 6 months. Participation in MB and post-intervention impacts varied for mothers with low versus high stress. The overall observed impacts were driven by positive impacts for mothers with low stress at baseline.

MB was well accepted and impactful for mothers with lower levels of stress but not for mothers with higher levels of stress. These findings support the value of identifying and differentiating sub-groups of at-risk families to better tailor services and improve outcomes. Specifically, programs may explore mechanisms to make it easier for mothers with higher levels of stress to participate in MB and to refine MB to be more effective for this group. The study identifies aspects of home visiting service and implementation systems that could be strengthened to better serve sub-groups of mothers. The attenuation of psychosocial impacts at 6 months and the differences in outcomes by baseline maternal stress underscores the need for ongoing investment and collaboration among home visiting programs, networks, and researchers to advance the field.
